# Delayed Diagnosis of Glutaric Aciduria Type 1: A Case Report

**DOI:** 10.7759/cureus.86380

**Published:** 2025-06-19

**Authors:** Cesar E Larancuent, Tracey Weiler, Sajel L Kana

**Affiliations:** 1 Health Sciences, Florida International University, Herbert Wertheim College of Medicine, Miami, USA; 2 Genetics, Florida International University, Herbert Wertheim College of Medicine, Miami, USA; 3 Genetics, Nicklaus Children's Hospital, Miami, USA

**Keywords:** biochemical marker, diagnostic testing, genetic disease, genetic testing, glutaric aciduria, low excretor phenotype, metabolic disorder, newborn screening, pediatric genetics, whole exome sequencing

## Abstract

Newborn screening (NBS) is performed to screen for conditions where early intervention can make a difference in a patient’s prognosis. We present the case of a patient with glutaric aciduria type 1 (GA1) that was missed on NBS but was diagnosed through exome sequencing (ES) at eight years of age. We report the case of a patient who was born in Florida in 2011 and underwent routine NBS, which was negative for all tested conditions, including amino acidemias. At 11 months of age, the patient presented with seizures. Upon physical examination, she had hypotonia, ptosis, and macrocephaly. A urinary tract infection was found, and the seizures were attributed to fever from an infectious source. Laboratory testing revealed marginally elevated levels of pyruvate, acylcarnitine, and total carnitine. Magnetic resonance imaging and spectroscopy (MRI/MRS) showed signs of basal ganglia damage. At 12 months of age, she was tested for pathogenic variants in PDHA1, PTEN, and the mitochondrial genome, to look for an underlying molecular diagnosis for her symptoms. No pathogenic variants were found. At eight years of age, ES revealed that the patient was a compound heterozygote for pathogenic variants in GCDH, confirming a diagnosis of GA1. There is little correlation between biochemical markers and phenotype severity in GA1; therefore, biochemical markers can potentially be within normal limits in a symptomatic patient. Diagnosis is complicated by low excretor phenotypes: some patients excrete lower levels of organic acids in the urine. Furthermore, there are no pathognomonic clinical findings, and these patients do not always have MRI findings. ES of our patient led to the diagnosis of GA1, where biomarkers and imaging did not, highlighting the clinical utility of gene sequencing. As we will explore, this is not an isolated case of disease missed in biochemical testing. GA1 should be included in a differential if a patient has symptoms consistent with the condition and/or if biochemical markers are marginally normal. Lowering the threshold for positive biochemical results is not sufficient to address this issue, as it would create an excess of false positives. Since GA1 can also result in inconclusive biochemical tests, false-negative results will still occur. As NBS cannot unequivocally rule out GA1 in these patients, enzyme and/or genetic testing may yield a diagnostic result and inform appropriate management.

## Introduction

Newborn screening (NBS) is a public health measure aimed at detecting many severe conditions amenable to management with diet and supplementation. These conditions are not readily observable in a clinical setting during the first days of life. Along with hearing tests, sweat chloride tests, and pulse oximetry, these screenings primarily test for hormonal or metabolic imbalances. NBS tests are highly sensitive, to ensure that newborns affected with a condition can be identified early enough for timely intervention. However, high sensitivity does result in the identification of false positives. High specificity is also required, as false positives can be stressful and costly and can lead to overtreatment [1]. However, some loss in specificity, leading to false positives, is tolerated to maximize sensitivity. Positive results on NBS are followed up with diagnostic testing to confirm the diagnosis.

This paper explores the case of a patient with glutaric aciduria type 1 (GA1) (OMIM#231670) that was missed on NBS. Glutaric aciduria type 1 (GA1) is caused by pathogenic variants in *GCDH*, which leads to deficiencies in glutaryl-CoA dehydrogenase (GCDH), an enzyme responsible for the breakdown of lysine, hydroxylysine, and tryptophan. Deficiency of this enzyme results in accumulation of these amino acids - and their metabolites - in the serum, resulting in brain damage, often manifesting clinically as macrocephaly and neurologic impairment [2]. This metabolic injury is particularly notable in the basal ganglia, responsible for controlling movement [2]. Affected individuals may also experience spasms, rigidity, or decreased muscle tone [2]. GA1 varies greatly in severity. Infections and associated fever, along with other high metabolic demand states that increase catabolism, can worsen the signs and symptoms of GA1. Conversely, dietary changes (protein restriction and carnitine supplementation) can help slow down the progression and impact of GA1 [2].

Because GA1 is a metabolic disease that is not clinically apparent at birth, and can be managed with dietary restrictions and close attention to fevers, it is included in NBS programs. Without biochemical testing, it is often undiagnosed as it has a latency period during which phenotypic changes are subtle as metabolites accumulate, ranging from a few months to three years [3]. Although there is variation in GA1 phenotype, there is no known association between biochemical marker levels and severity of the phenotype [4]. Furthermore, there is no pathognomonic clinical sign or symptom that can be identified before irreversible neurological damage takes place [5].

NBS for GA1 is performed by analyzing dried blood spots with tandem mass spectrometry (MS/MS), looking for elevated glutaryl carnitine (C5DC), along with an elevated C5DC/palmitic acid (C16) ratio. If positive, this is followed by second-tier testing, analyzing urine with MS/MS and gas chromatography-MS (GC/MS). These second-tier tests are aimed at detecting elevated levels of organic acids, such as 3-hydroxyglutaric and glutaric acids in the urine [3,6].

Similar to the second-tier testing after a positive newborn screen, patients with a clinical suspicion of GA1 undergo first-line diagnostic testing: urinalysis with GC/MS. Tissue and body fluids, such as cerebrospinal fluid (CSF), can also be analyzed with GC/MS or MS/MS to detect elevated levels of C5DC and C5DC/palmitic acid ratio [3]. Positive results from these tests are confirmed using GCDH mutation analysis. If two pathogenic variants are found in trans, on both maternally and paternally inherited genes, then the diagnosis of GA1 is confirmed. If not, however, this is followed by GCDH enzyme activity analysis. If the GCDH activity is found to be low, then the diagnosis is biochemically confirmed [5].

Complicating the diagnosis, some individuals have a low-excretor (LE) phenotype. These individuals excrete fewer organic acids into their urine. Because GA1 is diagnosed using levels of organic acid in urine, it is more difficult to diagnose GA1 in low excretor phenotypes [7]. Individuals with GA1 can exhibit normal levels of urinary organic acids, despite carrying pathogenic variants in both alleles of GCDH and having high levels of serum organic acids. LE GA1 has been defined as “molecular and/or clinical diagnosis of GA1 including biochemical data with a urinary glutaric acid excretion of <100 mmol/mol creatinine” [8,9]. It is estimated that 30-40% of individuals with GA1 have a LE phenotype [8]. The epistatic effect of the LE phenotype is also evident for other organic acidurias, including mitochondrial trifunctional protein (MTP) deficiency, long-chain 3-hydroxyacyl-CoA dehydrogenase (LCHAD) deficiency, and others [10]. A decrease in urinary organic acids is not correlated with decreased neurological damage nor an improved prognosis [5]; therefore, it is important to be aware of this possibility when working through a diagnostic work-up for patients with clinical signs of organic acidurias.

## Case presentation

We present the case of a Hispanic female born in Florida in 2011. Her NBS (Table 1) was negative for all tested conditions, including amino acidemias [11]. At 11 months of age, she was admitted to the hospital for seizures and encephalitis. On physical examination, she was found to have hypotonia, ptosis, and macrocephaly. Laboratory studies revealed a normal complete blood count (CBC), glucose, liver function testing (LFT), ammonia, phosphate, magnesium, and plasma free carnitine. On urinalysis (UA), the urine was found to be contaminated with more than 100,000 *Escherichia coli *bacterial colony-forming units. CSF was analyzed and found to be clear and colorless, with glucose, protein, and red blood cells (2/mm^3^ RBCs) at normal levels, and no growth on culture. A significant number of white blood cells (8/mm^3^ WBCs) were found, with 2/mm^3^ being polymorphonuclear cells (PMNs). Blood pyruvic acids were elevated: 0.18 mmol/L (normal: 0.08-0.16). Plasma acylcarnitine and total carnitine were marginally elevated, at 20 nmol/L (normal: 7-19 nmol/L) and 69 nmol/L (normal: 38-68 nmol/L), respectively (Table 2). Chromosomal microarray was significant for an 857 kb maternally inherited microduplication of 20q11.21, with undetermined significance.

**Table 1:** Florida newborn screening – conditions (2011) A list of conditions tested.

Category	Condition
Organic Acid	Propionic Acidemia (PROP)
	Methylmalonic Acidemia (Methylmalonyl-CoA Mutase Deficiency) (MUT)
	Methylmalonic Acidemia (Cobalamin Conditions)
	Isovaleric Acidemia (IVA)
	3-Methylcrotonyl-CoA Carboxylase Deficiency (3-MCC)
	3-Hydroxy-3-Methylglutaric Aciduria (HMG)
	Holocarboxylase Synthetase Deficiency (MCD)
	Beta-Ketothiolase Deficiency (BKT)
	Glutaric Acidemia, Type I (GA-1)
Amino Acid	Argininosuccinic Aciduria (ASA)
	Citrullinemia, Type I (CIT-I)
	Maple Syrup Urine Disease (MSUD)
	Homocystinuria (HCY)
	Classic Phenylketonuria (PKU)
	Tyrosinemia, Type I (TYR I)
Endocrine	Primary Congenital Hypothyroidism (CH)
	Congenital Adrenal Hyperplasia (CAH)
Hemoglobin	Sickle Cell Disease (Hemoglobin SS Disease)
Other	Biotinidase Deficiency (BIOT)
	Critical Congenital Heart Disease (CCHD)
	Cystic Fibrosis (CF)
	Classic Galactosemia (GALT)
	Hearing Loss or Varying Hearing Levels
	Fatty Acid Oxidation
	Carnitine Uptake Defect (CUD)
	Medium-Chain acyl-CoA Dehydrogenase Deficiency (MCAD)
	Very Long-Chain Acyl-CoA Dehydrogenase Deficiency (VLCAD)
	Long-Chain L-3 Hydroxyacyl-CoA Dehydrogenase Deficiency (LCHAD)
	Trifunctional Protein Deficiency (TFP)

**Table 2:** Serum lab values Selected serum lab values at initial presentation.

Serum labs	Value	Normal	Unit
Pyruvic acid	0.18	0.08-0.16	mmol/L
Acylcarnitine	20	7-19	nmol/L
Total carnitine	69	38-68	nmol/L

During the initial admission at 11 months of age, a computer tomography (CT) scan was performed, and no intracranial lesions were found. Magnetic resonance imaging (MRI) and spectroscopy (MRS) were also performed and were significant for increased T2-weighted signal and restricted diffusion in the lentiform nuclei diffusely, and caudate nuclei bilaterally, left greater than right, indicative of basal ganglia damage. The MRS showed no elevation of lactate; additionally, N-acetylaspartate (NAA), creatine, and choline were all within normal limits. Repeat MRI/MRS one week after admission confirmed abnormal signals from the basal ganglia, which extended into the cerebral peduncle; MRS was significant for decreased NAA peak (NAA as a marker of neuron viability), but otherwise normal. Video-EEG monitoring (vEEG) noted electrographic seizure activity originating from the left centrotemporal area and leading to rightward head and eye movement, along with tonic expansion of the right upper extremity. Renal ultrasound and video fluoroscopy were normal.

At 12 months of age, the coding regions of the PDHA1 gene were sequenced, associated with autosomal recessive pyruvate dehydrogenase E1-alpha deficiency (OMIM #300502). The patient was found to be heterozygous for an intronic variant of uncertain significance (c.831+15c>t, intron 8, heterozygous). Additionally, aiming to rule out neurodegenerative derangements, sequencing of PTEN and a mitochondrial common mutation and deletion screening panel were performed. No significant variants were detected.

At 13 months of age, the patient had another MRI, revealing evolving lesions in the basal ganglia, but no new lesions. At this time, very long chain fatty acids (VLCFAs) in the serum were within normal limits. The patient was then lost to follow-up until seven years old.

At seven years of age, the patient continued to have seizures, and the MRI performed was significant for basal ganglia lesions not extending to any cerebral structures. Laboratory analyses performed at this time included serum ammonia, ceruloplasmin, carnitine, lactic acid, and plasma acylcarnitine, as well as UA, and molecular testing for early-onset dystonia type 1 (DYT1), all of which were normal.

As the patient approached her eighth birthday, exome sequencing was performed. The patient was found to be a compound heterozygote in trans for pathogenic variants in GCDH (autosomal recessive GA1; OMIM# 608801). The maternal allele was c.680G>C and p.R227P, and the paternal allele was c.1198 G>A and p.V400M. Laboratory analyses at that time revealed a normal CBC, basic metabolic panel (BMP), urinalysis, vitamin D3, and plasma carnitine levels. Her urine C5DC was elevated, at 0.18 nmol/mL (normal: 0-0.10 nmol/mL), and her plasma amino acid test revealed non-specific, mild elevations of several amino acids (aspartic acid: 14 umol/L, glutamic acid: 170 umol/L, taurine: 248 umol/L, and methylmalonic-carnitine: 0.02 umol/L) (Table 3). These biochemical results were consistent with a clinical diagnosis of GA1. As of May 2024, the two variants identified in this patient are known to be pathogenic in fewer than 20 patients [12].

**Table 3:** Amino acid labs Measured amino acids during the patient's visit at eight years old.

Amino Acid	Source	Value	Normal (8 y/o)	Unit
Glutarylcarnitine (C5DC)	Urine	0.18	0-0.1	nmol/L
Aspartic acid	Plasma	14	2.9-12.6	µmol/L
Glutamic acid	Plasma	170	32-140	µmol/L
Taurine	Plasma	248	11-120	µmol/L
Methylmalonic-carnitine	Plasma	0.02	<0.05	µmol/L

## Discussion

**Figure 1:**
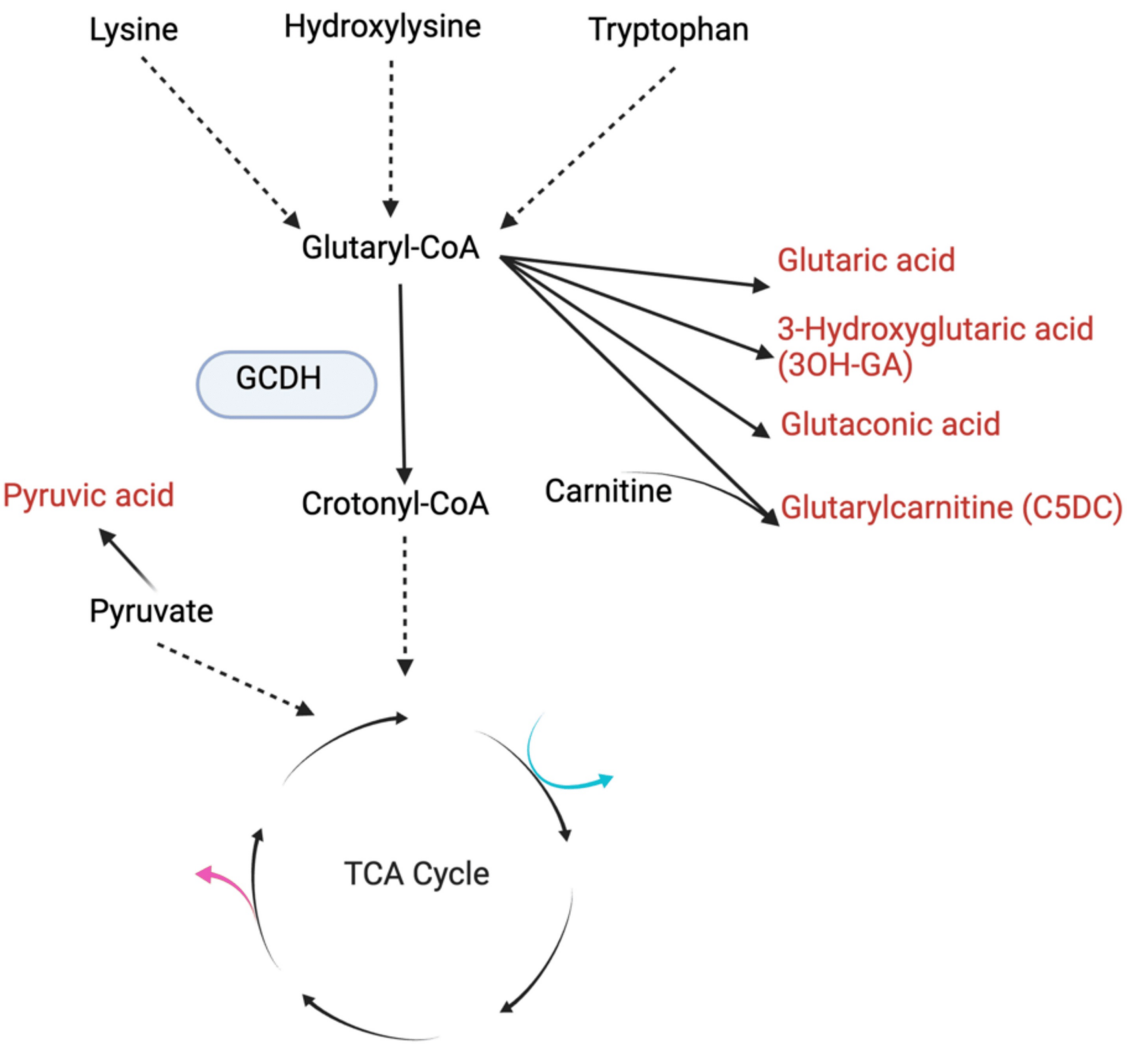
Breakdown of glutaryl-CoA by GCDH Breakdown of glutaryl-CoA by glutaryl-CoA dehydrogenase (GCDH), along with accumulated byproducts, including glutaric acid (GA). The tricarboxylic acid cycle is abbreviated as "TCA Cycle" in the figure. Image created by authors using BioRender.

The patient’s clinical presentation, characterized by seizures and encephalitis at 11 months, was consistent with GA1. Unfortunately, GA1 is challenging to diagnose as there is no clear diagnostic clinical sign for the condition. It was not until exome sequencing testing was performed, followed by sequencing of GCDH that she was diagnosed with GA1, highlighting the importance of genetic testing in establishing a diagnosis (biochemical breakdown of glutaryl-CoA illustrated in Figure 1).

The initial miss of the GA1 diagnosis on NBS sheds light on the limitations of such screening methods. NBS tests, such as the MS/MS of a dried blood spot (DBS) used in our patient’s case, are designed to cast a wide net - identifying all potential GA1 cases rather than providing a definitive diagnosis. While boasting a specificity of 99.42%, the sensitivity of this test stands at only 93.3% [3]. Consequently, reliance solely on NBS can lead to significant diagnostic oversights, as seen in this case and several others [13-17]. This diagnostic challenge is not unique to GA1; other metabolic diseases, such as MTP or LCHAD deficiencies, congenital adrenal hyperplasia (CAH), and carnitine palmitoyltransferase-II (CPT-II) deficiency, have also been missed on NBS [5,18-21], underscoring the need for more comprehensive diagnostic approaches.

In addition, when the patient was admitted to the hospital at 11 months of age, with clinical signs and symptoms, the diagnosis was missed during biochemical diagnostic testing. Because there is no association between the severity of the phenotype and biomarker levels, the patient’s serum biomarker levels were minimally elevated, despite significant clinical impact [4]. Furthermore, the fact that biochemical diagnostic testing is performed on urine samples can be complicated if the patient has an LE phenotype. She also had significant MRI findings, such as batwing appearance of sylvian features [22].

The LE phenotype results in reduced organic acid excretion in urine compared to individuals with a regular excretor phenotype, which can lead to unmet diagnostic thresholds for specific metabolites, consequently causing missed diagnoses [5]. When such a phenotype combines with marginally elevated serum levels, such as the slightly elevated blood pyruvic acid and plasma acylcarnitine observed in our patient, the biochemical diagnostic process can be particularly challenging. A search of the literature for other cases that shared the same pathogenic variants as our patient did not reveal any individuals described as LE. A paper published in 2021 looked at six individuals with LE variants, five of whom had normal NBS screens and one from outside the United States who did not have NBS [8]. Their biochemical profiles varied, with only 33% showing “semi-quantitative urine organic acid levels consistent with GA1 diagnosis” [8]. Notably, the GCDH variants in this study differed from our patient, emphasizing the diverse genetic landscape of this disorder and the complications it can present for diagnostic protocols.

Additionally, there have been reports in the past of affected individuals with “inconclusive biochemical abnormalities,” and for these patients, mutational analysis had been necessary to establish diagnosis [16], as it was with our patient. Further complicating matters, patients with metabolic disorders can sometimes have normal serum markers for their conditions' metabolites, even after being identified and confirmed through enzymatic or molecular testing. This has been seen in infants with trifunctional protein (TFP) deficiency and very long-chain acetyl-CoA dehydrogenase (VLCAD) deficiency [23].

**Figure 2:**
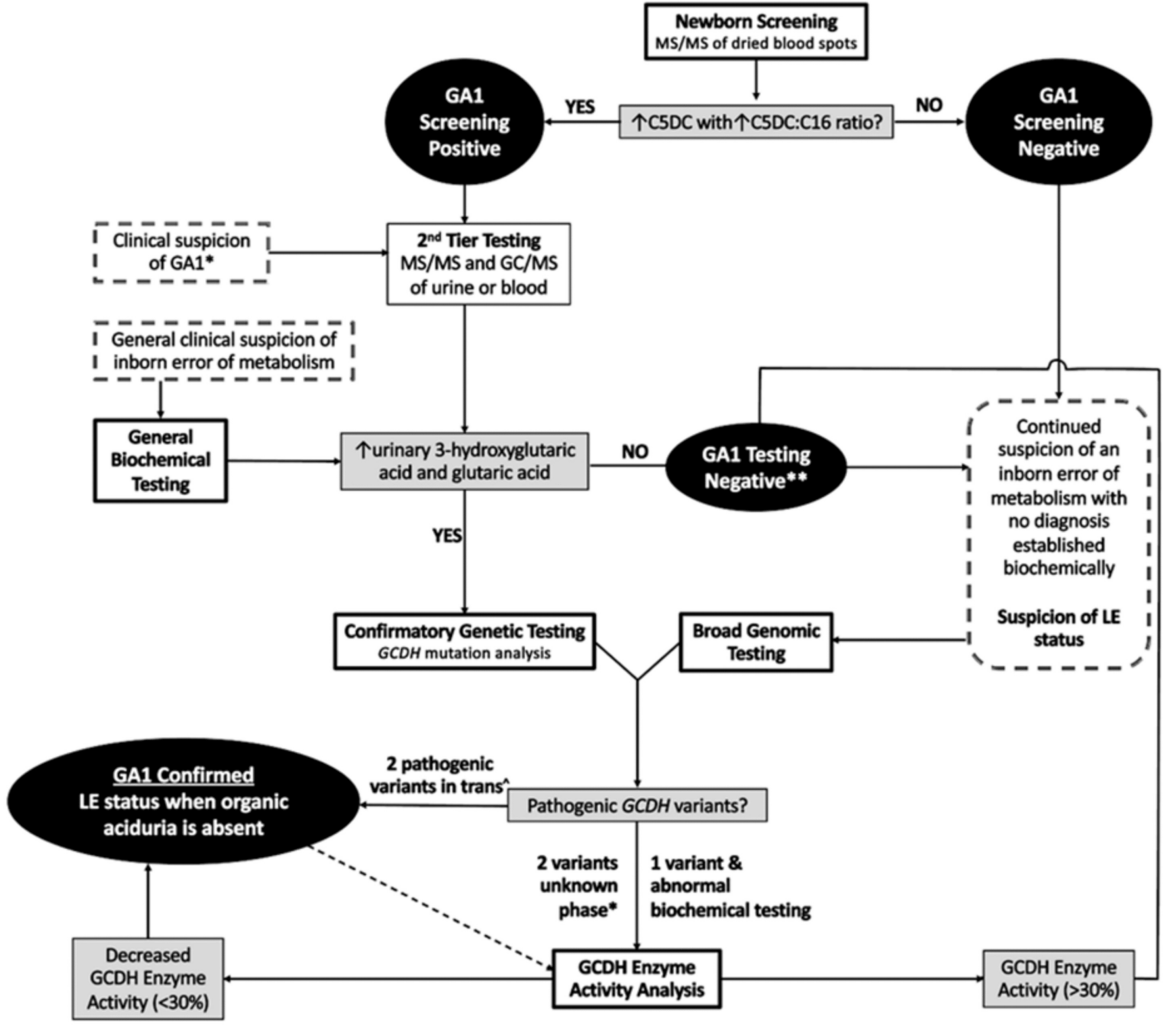
Diagnostic pathway of glutaric aciduria type 1 (GA1) Clinical suspicion of GA1* refers to clinical suspicion, regardless of screening status. 2 variants of unknown phase* refers to two variants found, but undetermined if they are in cis (in the same reference gene or DNA strand) or trans (in different DNA strands). GA1 Testing Negative**: GA1 testing is usually negative for one of two reasons - because the condition is not present or because the condition is present in a low excretor. MS/MS: tandem mass spectrometry. C5DC: glutarylcarnitine. C16: palmitic acid. GC/MS: gas spectrometry/mass spectrometry. GCDH: glutaryl-CoA dehydrogenase. Image credits: Authors

The ideal diagnostic pathway for GA1 is illustrated in Figure 2, along with integration of broad biochemical or genomic testing. To address the shortcomings of current biochemical testing methods, several reports have proposed strategies such as adjusting cutoffs based on gestational age, introducing second-tier screenings, and incorporating more specific tests, such as liquid chromatography-MS [4]. Specifically, for GA1, quantitative analysis in DBS of GA and 3OH-GA has been suggested [8]. However, the reliability of DBS in NBS remains a point of debate [1,19]. Advanced post-analytical tools, such as Mayo Clinic’s Collaborative Laboratory Integrated Reports (CLIR), can refine performance by considering a broader profile and additional ratios while adjusting for multiple variants, including age and weight [8].

## Conclusions

This case underscores the importance of considering GA1 in patients presenting with unexplained neurodegenerative symptoms, even in the setting of negative newborn screening results. It also highlights the potential limitations of NBS and the need for further diagnostic testing in cases where there is clinical suspicion of a metabolic disorder. Clinicians should maintain a high index of suspicion for GA1 and be aware of the potential for false-negative results in NBS, as well as how the existence of LE phenotypes may mask positive biochemical testing results in these patients, without offering any protection from pathological manifestations. Broad-based testing, particularly exome sequencing and the increasingly utilized genome sequencing, can be a critical diagnostic tool in identifying the causative genetic mutations in pediatric cases. As we move forward, genome sequencing is anticipated to surpass exome sequencing in its clinical use due to its ability to provide a more comprehensive genetic profile.

Engaging genetic specialists in this process can be highly beneficial, offering guidance on the selection of the most appropriate diagnostic tests, interpretation of results, and formulation of recommendations for management and ongoing care. This multidisciplinary approach ensures timely and accurate diagnosis, which can slow neurological decline and lead to improved patient outcomes. Increased awareness and education among clinicians regarding the presentation, diagnosis, and management of metabolic disorders such as GA1 is essential. By working together and utilizing the expertise of genetic specialists, clinicians can better navigate the complexities of diagnosing and managing patients with metabolic disorders, ultimately improving the quality of care provided to these patients.
